# Distinct immunomodulatory effects of synthetic progestins compared with natural progesterone in human immune cells

**DOI:** 10.1093/immhor/vlag046

**Published:** 2026-08-17

**Authors:** Apurva J Patel, Ankur N Karandikar, Aracely Miron-Ocampo, Nitin J Karandikar, Ashutosh K Mangalam

**Affiliations:** Department of Pathology, University of Iowa, Iowa City, IA, United States; Department of Pathology, University of Iowa, Iowa City, IA, United States; Department of Biology, College of Liberal Arts and Sciences, University of Florida, Gainesville, FL, United States; Department of Pathology, University of Iowa, Iowa City, IA, United States; Department of Pathology, University of Iowa, Iowa City, IA, United States; Department of Pathology, University of Iowa, Iowa City, IA, United States; Fraternal Order of Eagles Diabetes Research Center, University of Iowa, Iowa City, IA, United States

**Keywords:** cytokine, immunomodulation, IL-6, progesterone, synthetic progestins

## Abstract

Progesterone exerts important immunomodulatory functions, yet the immune effects of structurally diverse synthetic progestins remain incompletely defined. Given their variable receptor-binding profiles and links to altered inflammatory outcomes, we examined how commonly used progestins regulate immune signaling in primary human cells. Peripheral blood mononuclear cells from healthy female donors were exposed to physiologically relevant concentrations of natural progesterone (P4) and representative synthetic progestins spanning multiple generations. Cytokine responses were quantified at both the protein and transcript levels. Baseline steroid receptor expression and age of donor was correlated with cytokine output. High-dimensional flow cytometry coupled with unsupervised clustering was used to define the cellular sources of inflammatory cytokine production. In parallel, naïve CD4^+^ T-cell differentiation was performed to determine effects on T helper (Th) cell polarization. Only P4 induced immunoregulatory cytokine IL-10, whereas all progestins, except first-generation norethindrone, induced markedly higher IL-6 production compared with media control. Gene expression analysis confirmed induction of multiple proinflammatory genes at physiologically relevant concentrations. High-dimensional flow cytometry identified CD14^+^ monocytes as the dominant cellular source of IL-6 following progestin exposure. In contrast, P4 selectively suppressed Th1 differentiation and promoted Th2 lineage commitment, whereas synthetic progestins failed to reproduce these immunoregulatory effects. Together, these findings demonstrate that synthetic progestins differentially regulate innate and adaptive immune responses compared with P4 and promote proinflammatory responses that may influence immune homeostasis.

## Introduction

Progestogens are progesterone receptor (PR) ligands that include the natural hormone progesterone (P4) and a structurally diverse group of synthetic analogs known as progestins.^1^ Progestins are widely used in reproductive medicine for contraception, menopausal hormone therapy, and the management of gynecologic disorders such as endometriosis and abnormal uterine bleeding.^2–4^ Over several decades, multiple generations of progestins have been developed to improve potency, metabolic stability, and tolerability. These include first-generation estranes such as norethindrone, second-generation gonanes such as norgestrel and levonorgestrel, third-generation gonanes such as norgestimate, and fourth-generation progestins with distinct structural origins such as drospirenone.^5^^,^^6^ The present study focuses on representative progestins from each generation.

Although all progestins bind PR, they differ substantially in chemical structure and receptor binding profiles. Many synthetic progestins interact with additional steroid receptors, including the glucocorticoid receptor (GCR), androgen receptor (AR), and mineralocorticoid receptor (MR), which can lead to off-target biological effects.^7–10^ These pathways are highly relevant in immune cells, where steroid receptor signaling intersects with NF-κβ and other inflammatory regulators.^11^ As a result, synthetic progestins may produce immunological effects that differ from those of P4.

Progestin exposure has been associated with several immune-related and inflammatory conditions. Epidemiologic studies report increased risk of systemic lupus erythematosus,^12^ higher incidence of autoimmune diseases including multiple sclerosis (MS),^13^^,^^14^ and mood disturbances such as depression with the use of progestins.^15–17^ Progestins have also been linked to elevated risk of venous thrombosis,^18^^,^^19^ although in many of these studies the independent contribution of progestins cannot be fully separated from that of estrogen. Emerging reports from progestin-only exposure, including biopsy-confirmed ischemic colitis following progesterone-only use or norethindrone-only oral contraception,^20^ and cases of mesenteric or deep venous thrombosis linked to depot medroxyprogesterone acetate or levonorgestrel-only formulations,^21^ further suggest that progestins themselves may contribute to vascular and inflammatory pathways.

In addition, women who later developed MS were less likely to have become pregnant and more likely to fill prescriptions for hormonal preparations, including oral contraceptives, in the 5 years before the onset of typical MS symptoms. These patterns have been interpreted as behavioral changes occurring during the MS prodromal phase.^22^ Together, these observations highlight the importance of understanding how structurally distinct progestins influence immune pathways.

Despite their widespread use, the immunological effects of synthetic progestins remain poorly defined. Natural progesterone is generally considered immunomodulatory and can suppress inflammatory gene expression acting through nuclear PR.^11^ It is unknown whether commonly used synthetic progestins share these anti-inflammatory properties or whether structural differences and off-target receptor interactions result in proinflammatory activity. This gap in knowledge is significant given the global prevalence of progestin exposure and the increasing recognition of hormone-associated immune dysregulation.

Here, we investigated whether synthetic progestins differ from P4 in their ability to regulate inflammatory cytokine production in primary human immune cells. We examined whether widely used progestins act in a proinflammatory or anti-inflammatory manner in peripheral blood mononuclear cells (PBMCs) from healthy female donors.

## Materials and methods

### Primary cell preparation and steroid treatments

PBMCs from 42 healthy female donors aged 18 to 64 years and 6 male donors were isolated from deidentified leukocyte reduction system (LRS) cones containing leukocyte-rich whole blood from platelet donors at the University of Iowa DeGowin Blood Center. PBMC isolation was performed using Ficoll Paque (GE Healthcare) density gradient centrifugation, and cells were frozen in DMSO-containing media when not used immediately. Progesterone (P4) (Sigma-Aldrich P0130/5341) and synthetic progestins (norethindrone [Cayman Chemicals 20941; first generation], norgestrel [Cayman Chemicals 10006319; second generation], levonorgestrel [Cayman Chemicals 10006317; second generation], norgestimate [Cayman Chemicals, 16547; third generation], and drospirenone [Cayman Chemicals 23347; fourth generation]) were dissolved in absolute ethanol and stored as single-use aliquots in amber vials at −80 °C. PBMCs were thawed in RPMI media with DNase 10 KU/mL (Sigma-Aldrich, D4513-1vl) at 37 °C and plated at an optimized seeding density of 100,000 cells per well in 96-well plates^23^ in X-VIVO 15 serum-free media (Lonza, 04-418Q). Because physiological serum concentrations of progesterone and the maximum concentration (C_max_) values of most clinically used synthetic progestins fall within the 1- to 50-ng/mL range, we selected doses of 1, 10, and 50 ng/mL for in vitro stimulation. These doses approximate physiologic serum progesterone concentrations (1–35 ng/mL) and the reported C_max_ values of clinically used synthetic progestins, including norethindrone (4–10 ng/mL), levonorgestrel (6.7–39 ng/mL), norgestrel (0.6–2.6 ng/mL), norgestimate (0.6–2.5 ng/mL), and drospirenone (35–60 ng/mL).^24–28^ After 24 hours of exposure, supernatants were collected and concentrations of inflammatory cytokines (IL-6, TNF-α, IFN-γ, IL-1β, and GM-CSF) and anti-inflammatory cytokines (IL-10, IL-4, and TGF-β) were quantified by ELISA.

### ELISA

ELISA was performed on culture supernatants as per manufacturer protocol (BioLegend Human ELISA Kits for IFN-γ [430115], IL-6 [430501], TNF-α [430215], IL-1β [437015], GM-CSF [432004], IL-4 [430301], IL-10 [430604], and TGF-β [R&D Systems, DY240]). ELISA data were acquired on a BioTek Synergy H1 Hybrid Reader. Absorbance values were acquired using Gen5 software (BioTek) and exported for downstream statistical analysis.

### Quantitative PCR for cytokine gene expression

Levonorgestrel (second generation) and drospirenone (fourth generation) along with P4 were selected for gene expression analysis as these compounds showed robust induction of inflammatory cytokines in ELISA assay. Steroid-exposed PBMCs were harvested for RNA extraction using the Qiagen RNeasy Mini Kit. RNA quantity and purity were assessed using a NanoDrop spectrophotometer (Thermo Scientific), and 500 ng of total RNA was used for cDNA synthesis (Bio-Rad). Quantitative real-time PCR (qRT-PCR) reactions were prepared using Power SYBR Green PCR mastermix (Thermo Scientific, 4367659) and run on a StepOnePlus real-time PCR machine (Applied Biosystems). Human cytokine primers obtained from the Harvard Primer Databank using GeneIDs retrieved from NCBIgene. Sequences of all the primers used in the study are provided in Table S1. Cycle threshold (Ct) values were normalized to housekeeping genes (RPL and GAPDH), and relative gene expression was calculated using the ΔΔCt method with the media-only (no steroid) group as the reference control. Fold change values were derived using the 2^–ΔΔCt^ expression.^29^

### Intracellular cytokine staining

Bulk PBMCs exposed to steroids (P4, levonorgestrel, and drospirenone at 50 ng/mL) for 20 hours were treated with the protein transport inhibitor brefeldin A (Thermo Fisher) for an additional 4 hours. Following brefeldin A treatment, cells were washed twice with 1× PBS and once with FACS buffer, then surface stained with SparkBlue 574 CD3 (BioLegend, 300488), BUV496 CD19 (Thermo Fisher, B49619), BUV395 CD4 (BD Biosciences [BD], 564107), BUV565 CD8a (BD, 612914), BUV711 CD14 (BD, 563421), APC-eFluor CD123 (Thermo Fisher, 47-1239-42), and Live/Dead Blue viability dye (Thermo Fisher, L23105). For intracellular cytokine staining, cells were fixed and permeabilized using the eBioscience Fixation/Permeabilization Buffer Set, followed by incubation with RB780 IL-6 (BD, 624380) diluted in permeabilization buffer (Life Technologies). Samples were acquired on a Cytek Aurora flow cytometer, and data were analyzed using FlowJo software (version 10.10.1). Antibody panel design was guided by the FlowFinder tool (FluoroFinder), and fluorochrome selection was optimized to maintain a complexity score below 5 to minimize spectral overlap, based on the Cytek Aurora (Cytek Biosciences) marker design tool.

### High-dimensional analysis using t-SNE, X-shift clustering, ClusterExplorer, and traditional gating

High-dimensional single-cell analysis was performed to identify the immune cell subsets contributing to IL-6 production following progestin exposure. Compensated FCS files were exported from FlowJo and downsampled to an equal number of events per donor and treatment condition to prevent sampling bias.^30^ Downsampled datasets were imported into the analysis environment for unsupervised clustering using the X-shift algorithm, which groups cells based on multidimensional marker expression using a K-nearest neighbor density-estimation approach. The optimal K value was selected using the density-versus-K elbow plot. A t-distributed stochastic neighbor embedding (t-SNE) projection was then generated to visualize the high-dimensional structure of the PBMC compartment. X-shift cluster identities were overlaid onto the t-SNE map within ClusterExplorer to resolve major immune lineages and assess their spatial organization.

To determine the cellular sources of IL-6, cytokine-positive events were projected onto the t-SNE embedding and examined across X-shift clusters. ClusterExplorer was used to visualize IL-6 fluorescence intensity, marker expression profiles, and treatment-specific enrichment patterns across clusters. These analyses enabled identification of the immune cell subsets contributing to progestin-induced IL-6 production.

To validate findings from the unsupervised analysis, a traditional flow cytometry gating strategy was applied in parallel. After doublet exclusion and viability gating, major immune subsets were identified using canonical lineage markers, and IL-6^+^ cells were quantified specifically within the CD14^+^ monocyte population across all treatment conditions. This conventional gating approach served as an orthogonal method to confirm the identity of IL-6–producing cells and to compare cytokine responses across steroid exposures.

### Naïve CD4 T-cell differentiation

Naïve CD4^+^ T cells were negatively selected from freshly isolated PBMCs using the MojoSort Human CD4 Naïve T Cell Isolation Kit (BioLegend, 480042) according to the manufacturer’s instructions. Sorted CD4^+^ T cells were cryopreserved in DMSO-containing freezing media on the day of isolation for future use. For experiments, naïve CD4^+^ T cells were thawed in RPMI-1640 supplemented with DNase (10 KU/mL; Sigma-Aldrich, D4513-1VL), then resuspended at 1 × 10^6^ cells/mL in X-VIVO 15 serum-free medium (Lonza 04-418Q). Cells were preincubated for 24 hours with steroids: P4 (2 µg/mL), levonorgestrel (50 ng/mL), or drospirenone (50 ng/mL). P4 was used at a physiologically relevant concentration reflective of pregnancy-associated levels previously shown to exert anti-inflammatory effects.^31^ Levonorgestrel and drospirenone were used at 50 ng/mL, a dose selected based on ELISA and qPCR results indicating maximal cytokine induction. Because synthetic progestins exhibit substantially higher potency and progesterone-receptor affinity than P4, clinically relevant concentrations rather than equimolar matching were used to model real-world exposure scenarios. This design allows direct comparison of the immunological consequences of physiologically relevant P4 exposure versus contraceptive-range synthetic progestin exposure. After steroid pretreatment, cells were stimulated under distinct differentiation conditions (media/T helper [Th]0, Th1, Th2, Th17) as outlined in Table S2. Conditions were adapted from established protocol^32^ and included the following: media/Th0: no cytokines or neutralizing antibodies; Th1: anti-IL-4 (7 µg/mL; BD, 554481), IL-2 (10 ng/mL; BD, 554603), and IL-12 (10 ng/mL; BD, 554613); Th2: anti-IFN-γ (7 µg/mL; BD, 554698), IL-2 (10 ng/mL; BD, 554603), and IL-4 (10 ng/mL; BD, 554605); and Th17: anti-IL-4 (7 µg/mL; BD, 554481), anti-IFN-γ (7 µg/mL; BD, 554698), TGF-β1 (10 ng/mL; eBioscience, 14-8348-62), IL-1β (10 ng/mL; BD, 554602), and IL-6 (50 ng/mL; BD, 550071). Cells were activated with plate-bound anti-CD3 (1 µg/mL; eBioscience, 16-0037-85) and anti-CD28 (1 µg/mL; eBioscience, 16-0289-85) and incubated for 7 days at 37 °C. On day 7, supernatants were collected for ELISA-based quantification of IFN-γ (as mentioned before), IL-4 (BioLegend, 430301), and IL-17A (BioLegend, 433917) to assess polarization efficiency and steroid-specific effects on Th differentiation. Cells were washed twice with PBS and processed for intracellular cytokine staining as described in the “Intracellular cytokine staining” section above. Lineage-defining transcription factors and cytokines were used to evaluate Th1, Th2, and Th17A differentiation, including BV421 T-bet (BD, 563318), Alexa Fluor 488 IFN-γ (BD, 502510), APC GATA3 (BD, 560074), PE-Cy7 IL-4 (BD, 560783), PE-eFluor 610 RORγt (Thermo Fisher, 61698842), and BUV805-conjugated IL-17A (Thermo Fisher, A48291). Samples were acquired on a Cytek Aurora spectral cytometer, and data were analyzed using FlowJo version 10.10.1.

### Steroid receptor expression

Baseline steroid receptor expression was assessed in bulk PBMCs prior to hormone exposure. RNA was isolated from untreated cells and used for cDNA synthesis. Receptor transcript levels were quantified by qRT-PCR following the protocol described in the “Quantitative PCR for cytokine gene expression” section above. Primer sequences used for amplification are provided in Table S1. Fold change values were calculated relative to baseline expression in untreated PBMCs using the ΔΔCt method. To determine whether baseline steroid receptor expression predicted cytokine responsiveness, receptor mRNA levels measured under untreated conditions were correlated with cytokine output following hormone exposure on a donor-by-donor basis. Correlation analyses were performed separately for each receptor-hormone-cytokine combination using Spearman rank correlation. Correlation strength and statistical significance are reported, with *P* < 0.05 considered statistically significant. Donor age was evaluated as a potential source of interindividual variability in hormone-induced cytokine responses. Spearman correlation analyses were performed between age and cytokine secretion levels for each hormone condition. Age was treated as a continuous variable, and correlation coefficients and *P* values were calculated for all comparisons.

### Statistical analysis

GraphPad Prism (version 10.0.2) and R (version 4.3.1) were used for all statistical analyses. Data are expressed as median ± interquartile range. Because cytokine measurements and flow cytometry data were obtained from matched donors across multiple treatment conditions, statistical significance was primarily assessed using nonparametric tests. Repeated-measures comparisons were analyzed using the Friedman test with Dunn post hoc correction. When donor matching was not preserved, the Kruskal–Wallis test with Dunn correction was applied. More than 2 groups were compared via one-way ANOVA with a Bonferroni posttest for multiple comparisons. A *P* value <0.05 was considered statistically significant. R-based analyses and figure generation were performed using the tidyverse package suite, including ggplot2, dplyr, tidyr, readr, ggpubr, and patchwork. Spearman correlation analyses were performed using the cor.test() function, and multiple testing correction was performed using the Benjamini–Hochberg false discovery rate method with p.adjust(method = “BH”). Linear regression lines were added to correlation plots for visualization only and were not used for statistical inference.

### Study approval

All experiments were performed on PBMCs obtained from deidentified LRS cones from healthy platelet donors at the University of Iowa DeGowin Blood Center, as approved by the University of Iowa Institutional Review Board.

## Results

### Progestins induces higher IL-6 than progesterone in healthy female PBMCs

**Figure 1:**
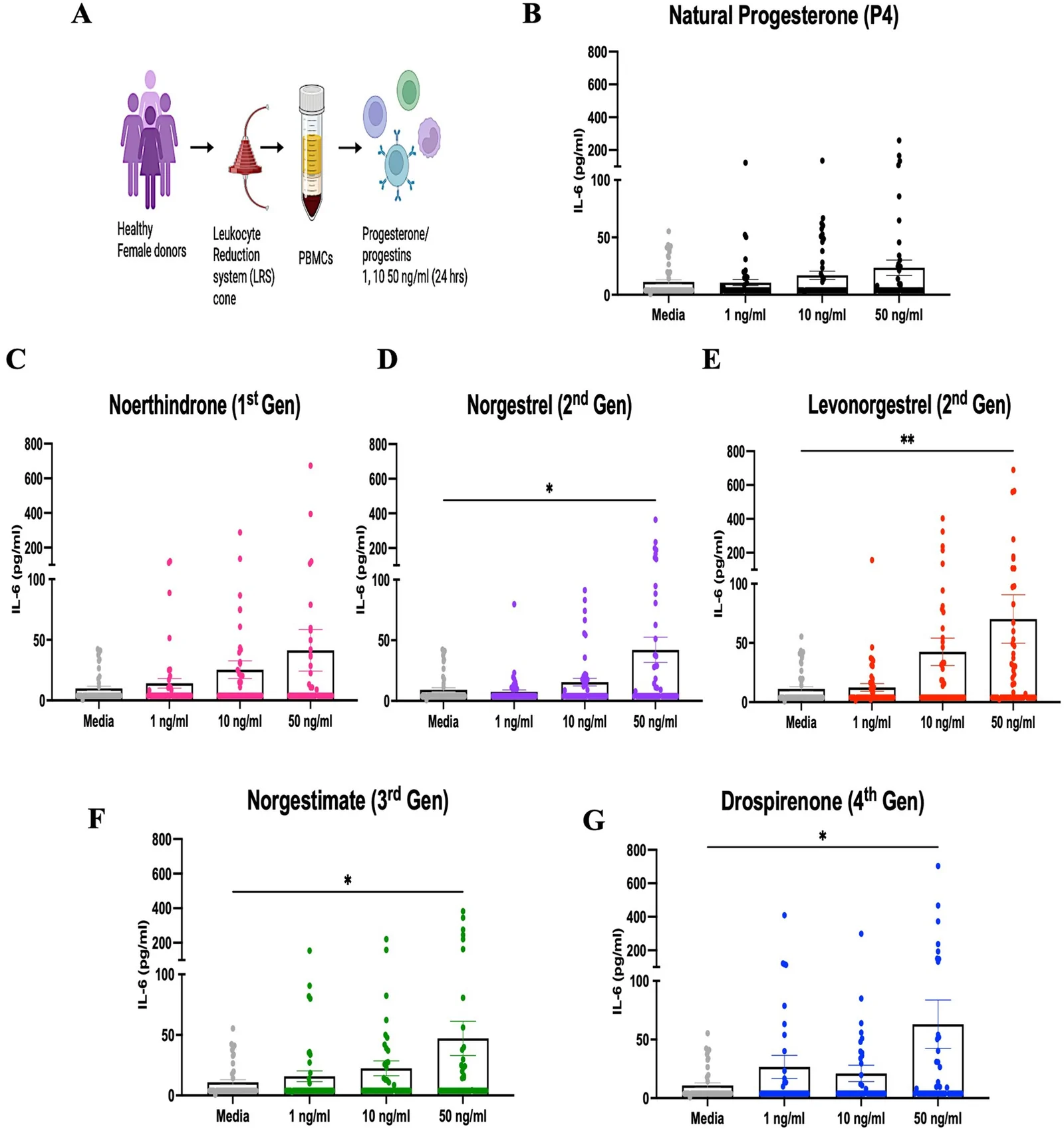
Synthetic progestins induce higher IL-6 production than natural progesterone (P4) in healthy female PBMCs. (A) Schematic overview of the experimental workflow. PBMCs were isolated from LRS cones obtained from 42 healthy female donors and treated for 24 hours with P4 or synthetic progestins at physiologically relevant concentrations (1, 10, or 50 ng/mL). (B–G) IL-6 concentrations measured by ELISA in culture supernatants following treatment with (B) P4, (C) norethindrone (first generation), (D) norgestrel (second generation), (E) levonorgestrel (second generation), (F) norgestimate (third generation), or (G) drospirenone (fourth generation), each compared with media control. Each data point represents an individual donor, and bars indicate median ± interquartile range. Statistical significance was assessed using the Friedman repeated-measures test with Dunn post hoc correction comparing P4/progestins with media control. Significant differences are indicated by asterisks (*P < 0.05, **P < 0.01).

As P4 exhibits immunomodulatory properties, we tested whether structurally distinct synthetic progestins differentially regulate inflammatory responses in human PBMCs. PBMCs were isolated from 42 healthy female donors and 6 healthy male donors using LRS cones obtained from the DeGowin Blood Center, University of Iowa. Preliminary titration assays determined that a concentration of 100 pg/mL of lipopolysaccharide was optimal for stimulating 10^5^ PBMCs per well, while conditions for T-cell receptor–independent activation using PMA/ionomycin were similarly standardized (Fig. S1A–C). Previous studies have shown that IL-6 and TNF-α rise rapidly within the first several hours of stimulation, whereas IL-1β and IFN-γ typically require prolonged activation and processing.^33–35^ These established kinetics guided the selection of a 24-hour exposure window. Furthermore, we utilized a concentration range of 1 to 50 ng/mL for all hormone treatments to mirror the physiological and pharmacological serum levels observed following oral ingestion in clinical settings (Fig. 1A).^5^^,^^36^^,^^37^

Upon applying these optimized conditions, we observed that synthetic progestins differentially modulated IL-6 production compared to P4. Natural progesterone produced modest, nonsignificant increases in IL-6 across some donors but overall remained substantially lower in magnitude than the responses induced by several synthetic progestins (Fig. 1B). Synthetic variants elicited more pronounced IL-6 elevations, although the magnitude and pattern of induction varied by compound and concentration. Because 10 donors exhibited IL-6 values below the lower limit of detection (LLOD), all samples were retained in the analysis by assigning LLOD-based values to nondetectable measurements to preserve group structure and avoid bias introduced by listwise exclusion. The first-generation progestin norethindrone showed no significant IL-6 elevation relative to media (Fig.  1C). In contrast, several synthetic progestins demonstrated significant increases in IL-6, particularly at higher concentrations. Among second-generation compounds, norgestrel significantly increased IL-6 at 50 ng/mL (Fig.  1D; *P* < 0.05), and levonorgestrel produced a stronger IL-6 response at the same dose (Fig.  1E; *P* < 0.01). The third-generation progestin norgestimate induced a modest but significant increase at 50 ng/mL (Fig.  1F; *P* < 0.05). The fourth-generation progestin drospirenone also elevated IL-6 at the highest concentration tested (Fig.  1G; *P* < 0.05).

Natural progesterone and synthetic progestins were also tested for their ability to stimulate PBMCs from male donors to provide a stable hormonal baseline and control for endogenous hormonal fluctuations. However, synthetic progestins did not induce any of the cytokines measured above baseline levels in male PBMCs (data not shown). Collectively, these results suggest that certain synthetic progestins have the ability to induce the proinflammatory cytokine IL-6 from healthy female PBMCs.

### Distinct cytokine profile induce by natural progesterone and synthetic progestins in female PBMCs

To determine how these compounds affect immune signaling more broadly, we quantified additional inflammatory and regulatory cytokines across all donors using limit of detection (LOD)–adjusted values for samples below the LLOD. All available samples were included in the analysis, and nondetectable values were substituted with LOD/2 to preserve donor structure and avoid exclusion-based bias. Natural progesterone produced small, nonsignificant increases in several proinflammatory cytokines but did not induce statistically significant elevations in IL-1β, GM-CSF, or TNF-α. In contrast, multiple synthetic progestins generated significant and more consistent increases in IL-1β and GM-CSF across donors.

**Figure 2:**
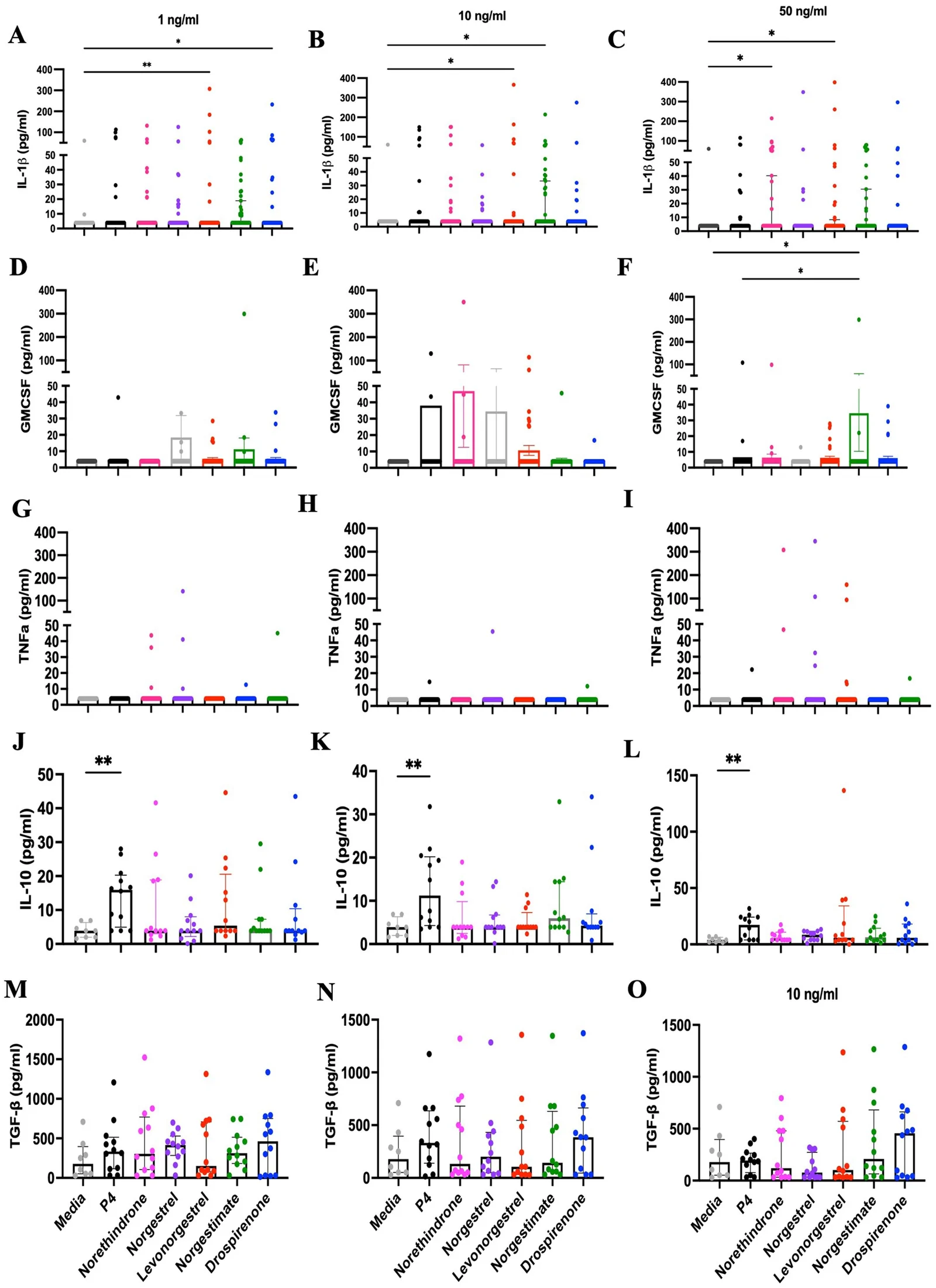
Distinct cytokines are induced by natural progesterone (P4) and progestins in healthy female PBMCs. PBMCs isolated from healthy female donors (*n* = 12–42) were treated for 24 hours with P4, norethindrone, norgestrel, levonorgestrel, norgestimate, or drospirenone at concentrations of 1, 10, or 50 ng/mL. Secretion of IL-1β, TNF-α, GM-CSF, IL-10, and TGF-β (pg/mL) was quantified in culture supernatants following treatment. IL-1β responses are shown at 1 ng/mL (A), 10 ng/mL (B), and 50 ng/mL (C); GM-CSF responses at 1 ng/mL (D), 10 ng/mL (E), and 50 ng/mL (F); TNF-α responses at 1 ng/mL (G), 10 ng/mL (H), and 50 ng/mL (I); IL-10 responses at 1 ng/mL (J), 10 ng/mL (K), and 50 ng/mL (L); and TGF-β responses at 1 ng/mL (M), 10 ng/mL (N), and 50 ng/mL (O). Each panel shows individual donor values with median ± interquartile range. Statistical comparisons were performed using the Friedman repeated-measures test with Dunn post hoc test, comparing all compounds with media. Significant differences are indicated by asterisks (*P < 0.05, **P < 0.01).

IL-1β showed the strongest and most consistent responsiveness to synthetic progestins. Levonorgestrel induced a robust and significant increase in IL-1β secretion at all 3 doses (Fig.  2A–C; *P* < 0.05), making it the most potent IL-1β–inducing compound in the panel. Norethindrone also elevated IL-1β, but only at the highest concentration of 50 ng/mL (Fig.  2C; *P* < 0.05), indicating a more restricted concentration-specific effect. Interestingly, drospirenone increased IL-1β at the lowest dose of 1 ng/mL (Fig.  2A; *P* < 0.05), although this effect did not persist at higher concentrations. Other progestins produced minimal or no IL-1β induction relative to media.

GM-CSF responses were more selective (Fig.  2D–F). Norgestimate significantly increased GM-CSF secretion at 50 ng/mL (Fig.  2F; *P* < 0.05), whereas the other progestins showed no meaningful elevation at any dose.

In contrast, TNF-α secretion remained largely unchanged across all treatments and doses (Fig.  2G–I). None of the synthetic progestins significantly increased TNF-α, indicating that TNF-α is not a major target of progestin-driven activation in PBMCs under these short-term conditions.

No detectable IFN-γ or IL-4 was observed after 24 hours of exposure to progesterone or synthetic progestins (data not shown), consistent with the known requirement for prolonged stimulation to activate these effector pathways. In contrast, P4 induced significantly higher IL-10 at all doses tested, whereas none of the synthetic progestins increased IL-10 production (Fig.  2J–L). TGF-β levels remained stable across all compounds and concentrations (Fig.  2M–O).

Overall, these results demonstrate that synthetic progestins are more likely than P4 to trigger proinflammatory cytokine production, particularly IL-1β, while lacking the ability to induce the anti-inflammatory cytokine IL-10. Based on their consistently elevated cytokine profiles, levonorgestrel (second generation) and drospirenone (fourth generation) were selected for further mechanistic analysis.

### Natural progesterone and synthetic progestins differentially regulate pro- and anti-inflammatory cytokine gene expression

To determine whether the elevated cytokine protein levels observed in our ELISA assays were driven by corresponding transcriptional changes, we quantified cytokine mRNA fold change in a subset of donor samples using qPCR. Levonorgestrel, a second-generation progestin, and drospirenone, a fourth-generation progestin, were selected for transcriptomic analysis because they produced the strongest cytokine induction at the protein level.

**Figure 3:**
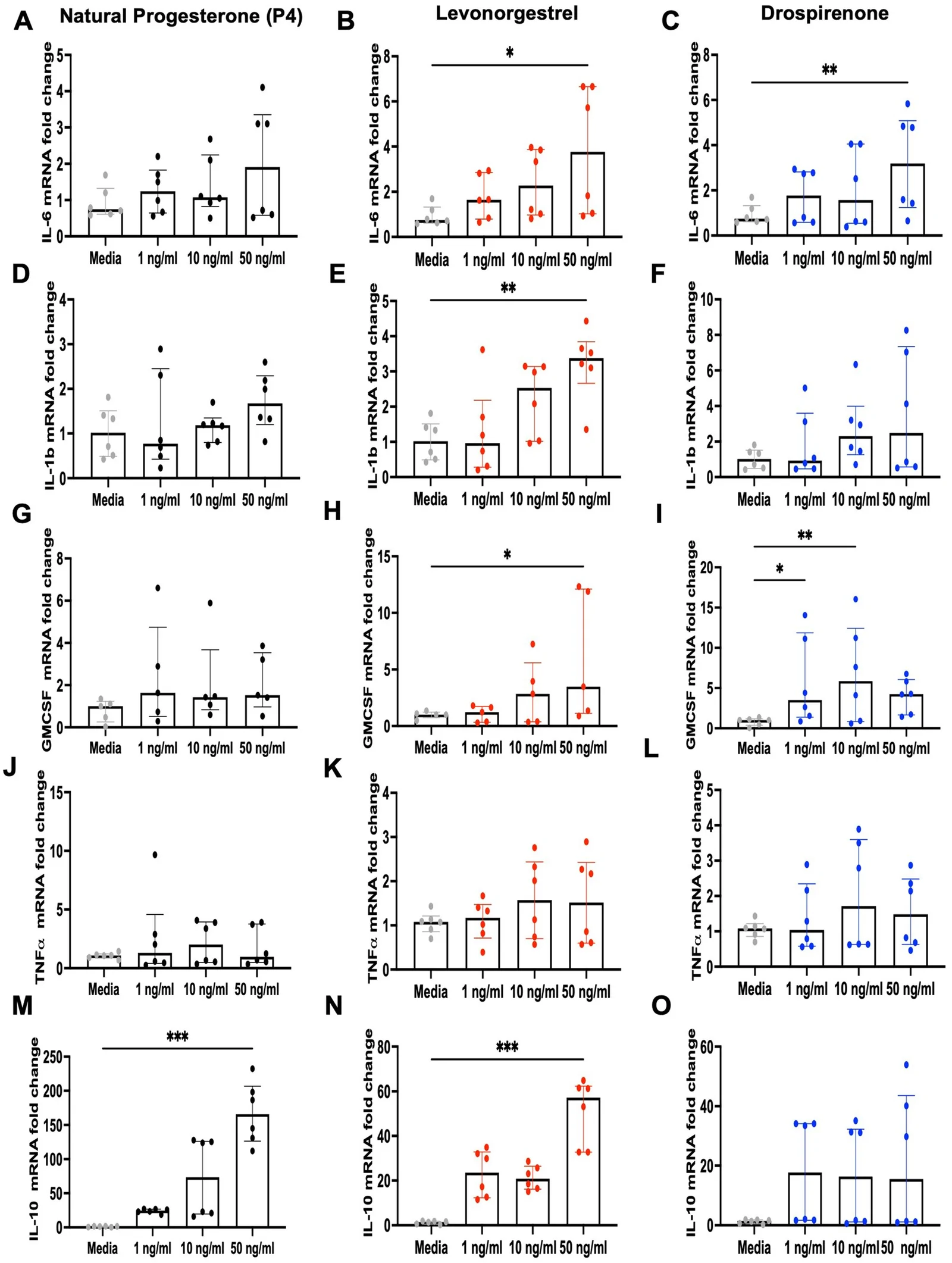
Distinct cytokine gene expression is induced by natural progesterone (P4) and synthetic progestins in healthy female PBMCs. (A–C) Fold change in IL-6 mRNA expression following treatment with P4 (A), levonorgestrel (B), or drospirenone (C) at concentrations of 1, 10, and 50 ng/mL. (D–F) Fold change in IL-1β mRNA expression following treatment with P4 (D), levonorgestrel (E), or drospirenone (F). (G–I) Fold change in GM-CSF mRNA expression following treatment with P4 (G), levonorgestrel (H), or drospirenone (I). (J–L) Fold change in TNF-α mRNA expression following treatment with P4 (J), levonorgestrel (K), or drospirenone (L). (M–O) Fold change in IL-10 mRNA expression following treatment with P4 (M), levonorgestrel (N), or drospirenone (O). Values were normalized with housekeeping gene controls (GAPDH), *n* = 6. Gene expression values were derived using ΔCt normalization, fold change was computed using the 2^–ΔΔCt^ method, and statistical differences between each progestin and P4 with media were compared using a Friedman repeated-measures test with Dunn post hoc pairwise comparisons. Significant differences are indicated by asterisks (*P < 0.05, **P < 0.01, *P < 0.001).

Natural progesterone exhibited a modest, nonsignificant increase in IL-6 transcript expression (Fig.  3A). In contrast, both synthetic progestins significantly increased proinflammatory cytokine transcripts compared with media controls. Levonorgestrel and drospirenone each induced a marked rise in IL-6 mRNA at the 50 ng/mL dose (Fig. 3B and C; *P* < 0.05), consistent with their ELISA profiles. Natural progesterone did not significantly alter IL-1β transcription (Fig.  3D). IL-1β mRNA was also significantly elevated by levonorgestrel at 50 ng/mL (Fig. 3E; *P* < 0.05), whereas drospirenone produced only a modest transcriptional increase that did not reach significance (Fig. 3F). This pattern mirrors the limited IL-1β protein induction observed in ELISA.

GM-CSF mRNA showed a more nuanced pattern. Natural progesterone did not significantly change GM-CSF transcript levels (Fig.  3G), whereas levonorgestrel significantly increased GM-CSF transcription at 50 ng/mL (Fig. 3H) (*P* < 0.05 and *P* < 0.01), whereas drospirenone induced significant GM-CSF upregulation at 1 and 10 ng per mL (Fig. 3I; *P* < 0.05 and *P* < 0.01). TNF-α mRNA remained unchanged across all doses for both compounds (Fig. 3J–L), consistent with the absence of TNF-α induction in ELISA.

Among regulatory cytokines, IL-10 mRNA was significantly upregulated at 50 ng/mL for P4 and levonorgestrel, with P4 producing the higher fold change, exceeding 200-fold (Fig. 3M–O). Notably, this transcriptional increase in IL-10 with levonorgestrel at 50 ng/mL was not reflected at the protein level by ELISA, suggesting posttranscriptional regulation, limited translation, or rapid consumption of secreted IL-10 in this short-term assay. Overall, these transcriptional data show that synthetic progestins elicit more consistent IL-6 and IL-1β gene induction than P4, while progesterone itself is not transcriptionally inert and can induce low-level proinflammatory and regulatory cytokine expression in some donors.

### Baseline steroid receptor expression and donor age do not predict cytokine responsiveness

**Figure 4:**
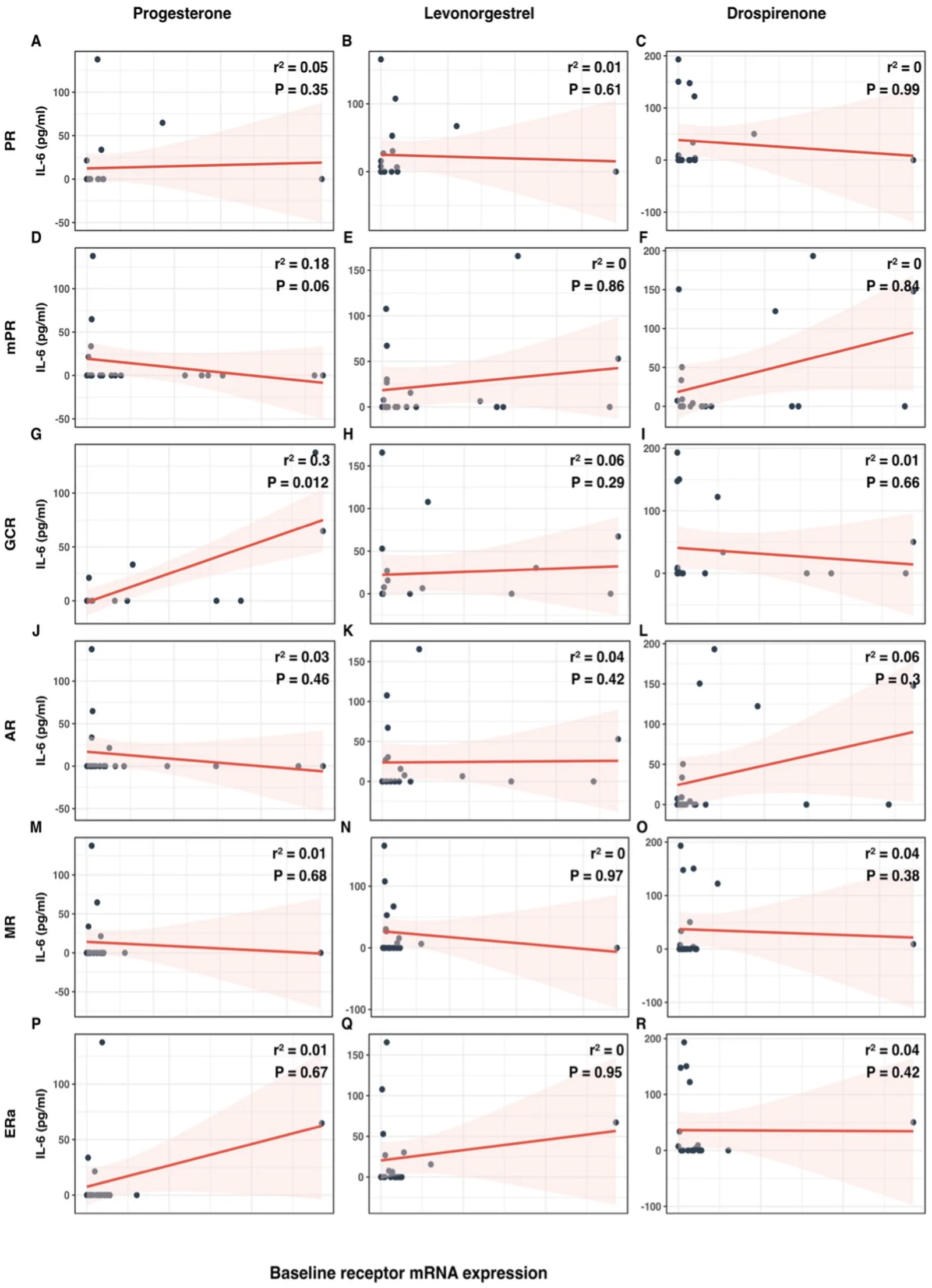
Correlation between baseline receptor mRNA expression and IL-6 production following progestin treatment. Baseline mRNA expression of progesterone receptor (PR), membrane progesterone receptor (mPR), glucocorticoid receptor (GCR), androgen receptor (AR), mineralocorticoid receptor (MR), and estrogen receptor alpha (ERα) was correlated with IL-6 secretion (pg/mL) after treatment with progesterone, levonorgestrel, or drospirenone (50 ng/mL). Each panel shows individual donor values with a fitted line and 95% confidence interval. Panels show correlations between PR expression and IL-6 secretion following hormone exposure: progesterone (A), levonorgestrel (B), and drospirenone (C). (D–R) Correlations for mPR (D–F), GCR (G–I), AR (J–L), MR (M–O), and ERα (P–R) are shown under the same treatment conditions. Reported statistics are Spearman correlation coefficients (ρ) with corresponding *P* values; the r^2^ values shown in the panels represent Spearman rho (ρ), *n* = 20.

To determine whether baseline receptor expression influences cytokine responsiveness to hormone exposure, we examined correlations between steroid receptor mRNA levels and cytokine secretion across progesterone and synthetic progestin treatments (Fig. 4A–R; Fig. S2–S4). Overall, baseline receptor expression showed limited predictive value for cytokine output.

For IL-6, PR expression did not significantly correlate with cytokine secretion under progesterone, levonorgestrel, or drospirenone treatment (Fig. 4A–C; all r^2^ ≤ 0.05, *P* > 0.3). Similarly, membrane progesterone receptor (mPR) expression showed no significant associations with IL-6 responses, although a modest trend was observed with progesterone treatment (Fig. 4D–F; progesterone: r^2^ = 0.18, *P* = 0.06). Among all receptors examined, GCR expression showed the strongest association with progesterone-induced IL-6 secretion (Fig. 4G; r^2^ = 0.30, *P* = 0.012). However, this association appeared to be influenced by a high-value donor sample, and therefore the finding should be interpreted cautiously. No comparable associations between GCR expression and IL-6 secretion were observed for levonorgestrel or drospirenone (Fig. 4H and I; all *P* > 0.29). AR, MR, and estrogen receptor alpha (ERα) expression likewise showed no significant relationships with IL-6 secretion across treatments (Fig. 4J–R; all r^2^ ≤ 0.06, *P* > 0.3). Collectively, these findings indicate that baseline receptor expression does not robustly predict IL-6 responsiveness to progesterone or synthetic progestins.

We next examine whether baseline receptor expression predicted IL-1β responsiveness following progesterone, levonorgestrel, or drospirenone exposure (Fig. S2A–R). For all 3 hormones, IL-1β production did not correlate with expression of PR, mPR, GCR, AR, MR, or ERβ, indicating that baseline receptor levels do not explain donor-to-donor variability in IL-1β responses.

We next assessed whether baseline receptor expression influenced GM-CSF secretion (Fig. S3A–R). Under progesterone treatment, modest but significant associations were observed between GM-CSF secretion and mPR (Fig. S3D; r^2^ = 0.24, *P* = 0.027), AR (Fig. S3J; r^2^ = 0.23, *P* = 0.033), and MR (Fig. S3M; r^2^ = 0.24, *P* = 0.029) expression. No significant associations were observed for PR, GCR, or ERβ expression in the progesterone-treated group. Levonorgestrel treatment did not show any significant correlations between baseline receptor expression and GM-CSF secretion (all *P* > 0.05), although several modest trends were observed. In contrast, drospirenone-induced GM-CSF secretion showed significant positive associations with PR (Fig. S3C; r^2^ = 0.21, *P* = 0.04), GCR (Fig. S3I; r^2^ = 0.21, *P* = 0.04), and AR (Fig. S3L; r^2^ = 0.27, *P* = 0.019) expression. No significant associations were observed for mPR, MR, or ERβ expression under drospirenone treatment.

For TNF-α, baseline receptor expression showed minimal association with cytokine output across treatments (Fig. S4A–R). No significant correlations were observed under progesterone or levonorgestrel exposure. Under drospirenone treatment, GCR expression demonstrated a modest positive correlation with TNF-α secretion (Fig. S4I; r^2^ = 0.21, *P* = 0.044). However, given the limited sample size and the absence of similar relationships across other treatment conditions, this finding should be interpreted cautiously. Together, these findings indicate that baseline steroid receptor expression provides limited explanatory power for cytokine responsiveness, with only isolated receptor–cytokine associations emerging under specific hormone conditions.

**Figure 5:**
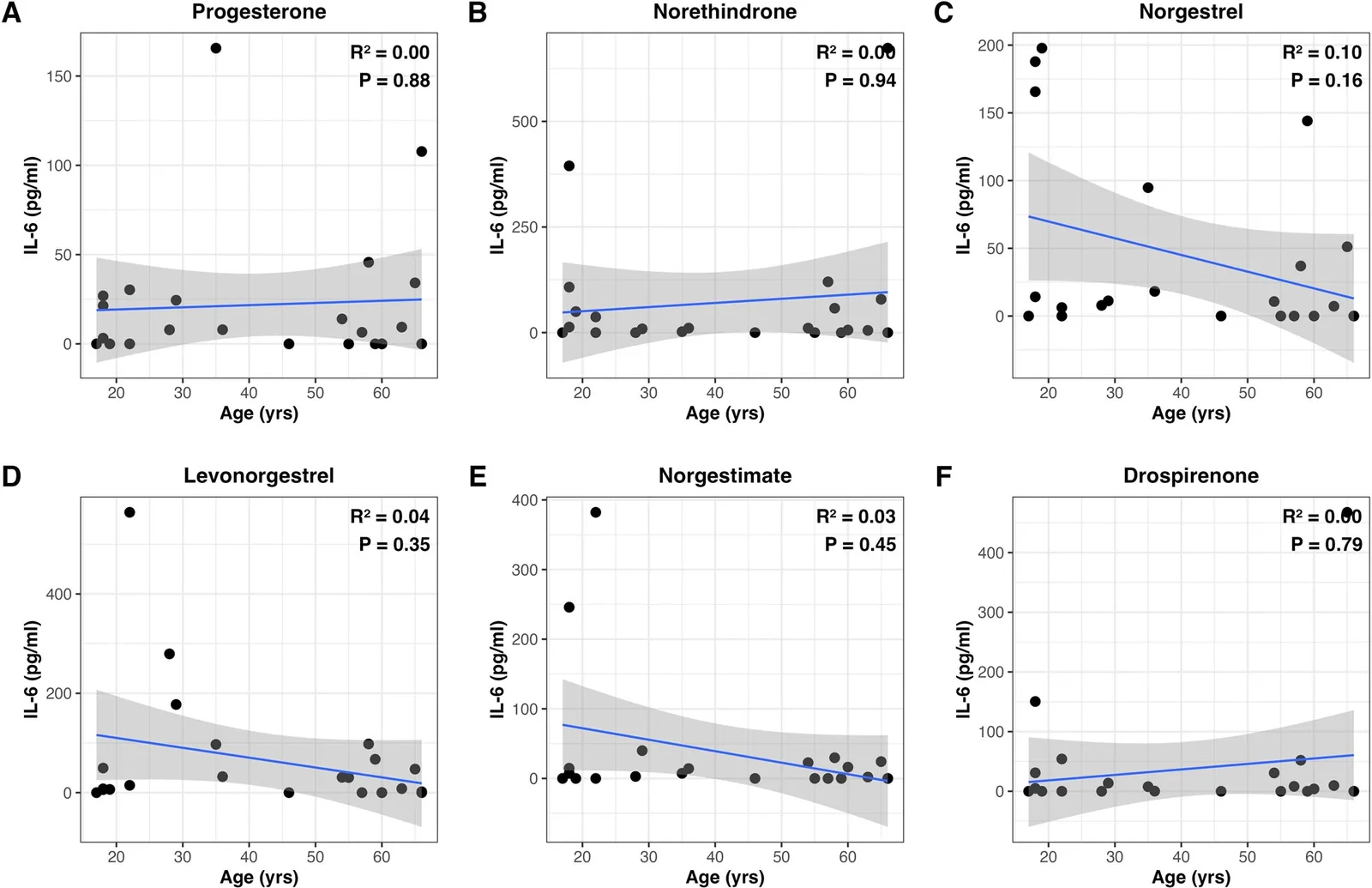
Relationship between age and IL-6 production following progestin treatment. Age (years) of PBMC donors was correlated with IL-6 secretion (pg/mL) measured in culture supernatants following treatment with (A) progesterone, (B) norethindrone, (C) norgestrel, (D) levonorgestrel, (E) norgestimate, or (F) drospirenone. Each panel shows individual donor values with a fitted line and 95% confidence interval. Reported statistics are Spearman correlation coefficients (ρ) with corresponding *P* values, and the r^2^ values shown in the panels represent Spearman rho (ρ), *n* = 20.

To determine whether donor age contributed to variability in IL-6 responses, we examined correlations between age and IL-6 secretion across all hormone treatments (Fig. 5A–F). Age showed no significant relationship with IL-6 levels under progesterone, norethindrone, levonorgestrel, drospirenone, or norgestimate exposure, with all r^2^ values ranging from 0.00 to 0.04 and all *P* values >0.35. A modest, nonsignificant trend was observed for norgestrel (Fig. 5C, r^2^ = 0.10, *P* = 0.16), but this did not reach statistical significance. Age likewise did not significantly correlate with IL-1β, GM-CSF, or TNF-α levels for any progestins (Fig. S5A–R). Overall, these findings indicate that age does not meaningfully influence IL-6 responsiveness to natural or synthetic progestins in this donor cohort.

### CD14^+^ monocytes are the dominant IL-6–producing population across progestin treatments

**Figure 6:**
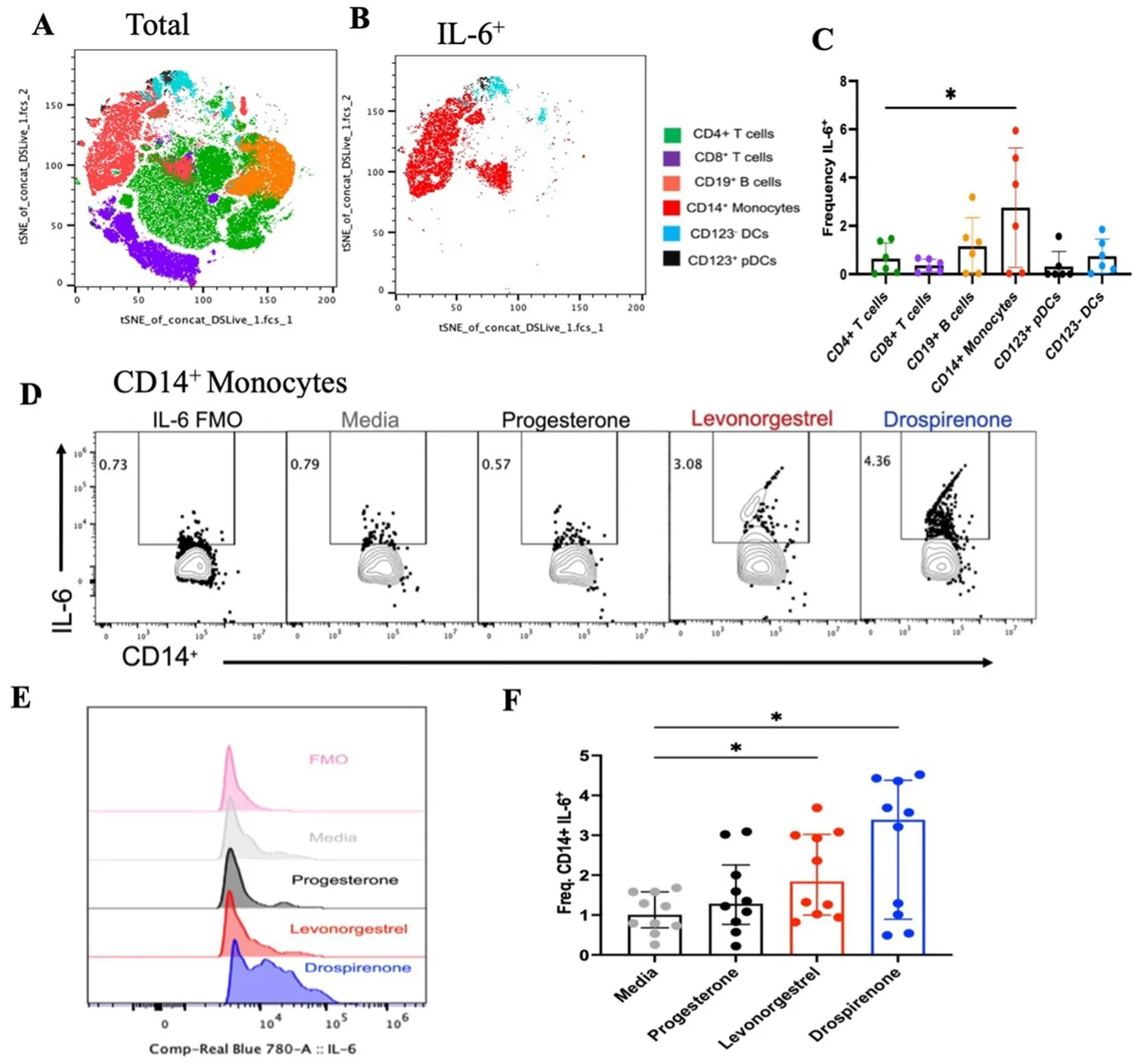
CD14^+^ monocytes are the dominant IL-6–producing population across progestin treatments. (A) t-SNE map of downsampled PBMCs showing distinct X-shift immune clusters. Cluster identities were assigned using ClusterExplorer based on lineage marker expression, including CD4^+^ T cells, CD8^+^ T cells, CD19^+^ B cells, CD14^+^ monocytes, CD123^−^ conventional dendritic cells (DCs), and CD123^+^ plasmacytoid dendritic cells (pDCs). (B) IL-6^+^ events projected onto the same t-SNE embedding, highlighting IL-6-enriched clusters localized primarily within monocyte, pDC, and DC regions. (C) Frequency of IL-6^+^ cells across major immune lineages, demonstrating selective enrichment of IL-6 expression within CD14^+^ monocytes, CD123^−^ DCs, and CD123^+^ pDCs. Bars represent median ± interquartile range; *P < 0.05. (D) Representative flow cytometry plots showing IL-6 expression within gated CD14^+^ monocytes across media, progesterone, levonorgestrel, and drospirenone treatment conditions, with fluorescence-minus-one (FMO) control shown for reference. (E) Overlay histograms of IL-6 fluorescence in CD14^+^ monocytes illustrating increased IL-6 signal intensity following levonorgestrel and drospirenone exposure. (F) Frequency of CD14^+^ IL-6^+^ monocytes across donors. Each point represents an individual donor; lines indicate paired samples. Bars represent median ± interquartile range; *P < 0.05. Statistical comparisons were performed using the Friedman test with Dunn post hoc correction. A *P* value <0.05 was considered statistically significant.

Since IL-6 was strongly induced by levonorgestrel and drospirenone at both the protein and mRNA levels, we utilized intracellular flow cytometry to identify the immune subsets responsible for IL-6 production. To map the cellular origins of IL-6 following progestin exposure, a t-SNE projection of downsampled PBMCs was generated, and X-shift clustering was performed within ClusterExplorer, identifying 17 phenotypically distinct immune cell clusters based on multidimensional marker expression (Fig. 6A). This unsupervised analysis resolved major immune lineages, including CD14^+^ monocytes, CD19^+^ B cells, CD123^+^ dendritic cells, CD4^+^ and CD8^+^ T cells, and CD123^+^ plasmacytoid dendritic cells, which were visualized by overlaying cluster identities onto the t-SNE embedding. Projection of IL-6 expression onto the same t-SNE map identified IL-6-enriched clusters, indicating that progestin-induced cytokine production was restricted to discrete subsets rather than broadly distributed across all PBMC populations (Fig. 6B). Lineage mapping revealed that these IL-6^+^ clusters were predominantly composed of CD14^+^ monocytes, CD123^−^ conventional dendritic cells, and CD123^+^ plasmacytoid dendritic cells, with monocytes representing the largest contributor. Quantitative analysis confirmed this pattern, showing significantly increased frequencies of IL-6^+^ cells in drospirenone-treated PBMCs by CD14^+^ monocytes (Fig. 6C). To validate the findings from the high-dimensional clustering approach, a traditional flow cytometry gating strategy was applied (Fig. S6). Using forward and side scatter parameters and lineage-defining surface markers, IL-6^+^ cells were quantified specifically within the CD14^+^ monocyte compartment across all treatment conditions, including media, P4, levonorgestrel, and drospirenone (Fig. 6D). Overlay histograms revealed a clear rightward shift in IL-6 fluorescence intensity following levonorgestrel and drospirenone exposure, indicating enhanced per-cell cytokine production (Fig. 6E).

The frequency of CD14^+^ IL-6^+^ cells differed significantly across treatments, with levonorgestrel and drospirenone eliciting the strong response as compared to P4 (Fig. 6F). Together, these complementary analyses, spanning high-dimensional clustering, lineage-resolved visualization, and conventional gating, demonstrate that CD14^+^ monocytes are the dominant IL-6–producing population following progestin exposure. Additionally, our data showed that drospirenone and levonorgestrel consistently drive the strongest activation among the tested compounds.

### Natural progesterone, but not synthetic progestins, selectively modulates Th1 and Th2 differentiation

**Figure 7:**
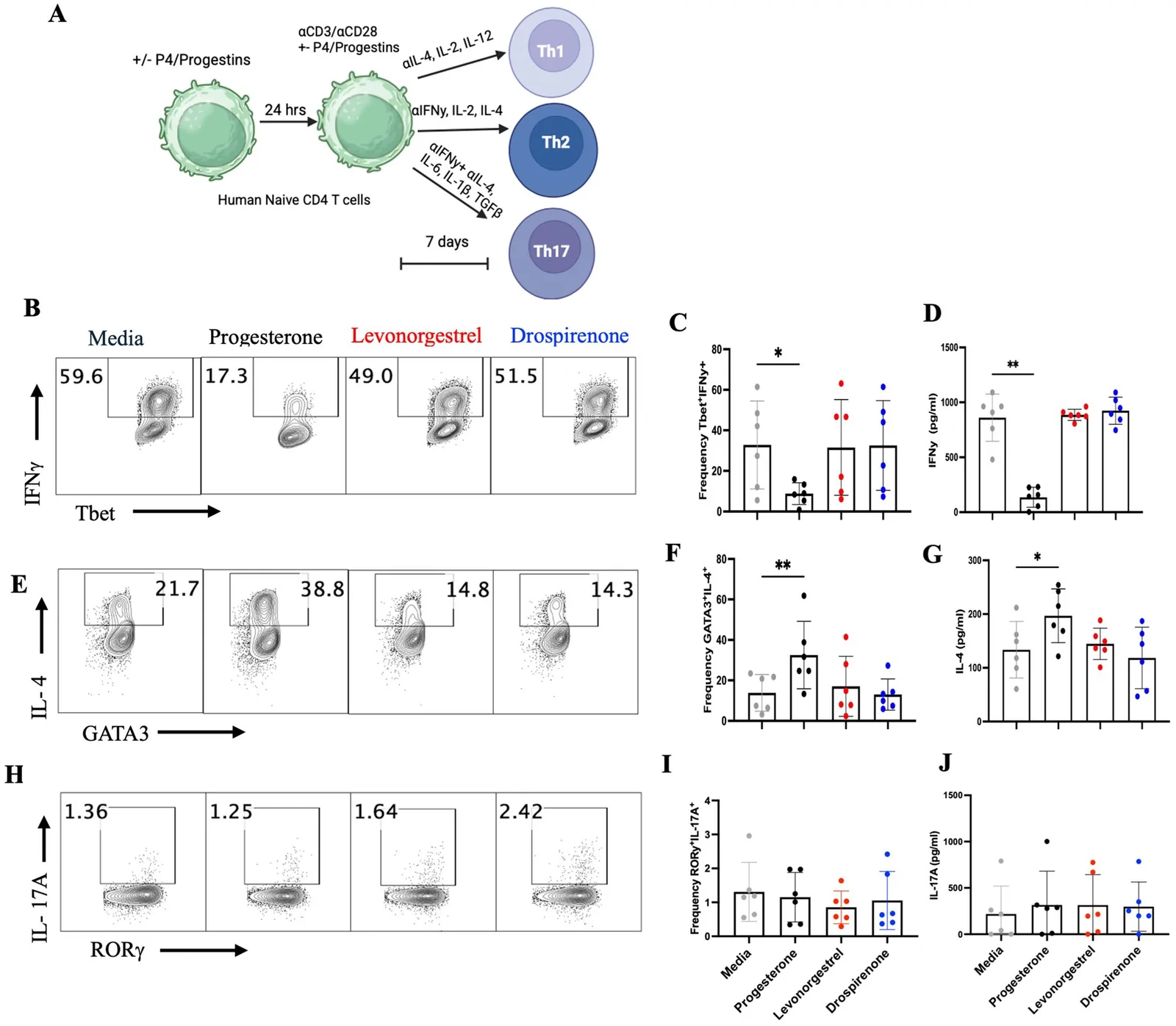
Natural progesterone (P4), but not synthetic progestins, directs T-cell polarization. (A) Schematic of the experimental workflow for the differentiation of naïve CD4^+^ T cells into Th1, Th2, and Th17 lineages following steroid pretreatment. (B) Representative flow cytometry plots showing T-bet^+^IFN-γ^+^ expression in CD4^+^ T cells under Th1-polarizing conditions. (C) Summary bar graphs showing the frequency of T-bet^+^ IFN-γ^+^ cells. (D) IFN-γ concentrations in matched culture supernatants measured by ELISA. (E) Representative flow cytometry plots showing GATA3^+^IL-4^+^ under Th2-polarizing conditions, with (F) bar graphs quantifying GATA3^+^ IL-4^+^ cells. (G) IL-4 concentrations in matched culture supernatants measured by ELISA. (H) Representative flow cytometry plots showing RORγt^+^IL-17A^+^ expression under Th17-polarizing conditions, with (I) bar graphs quantifying RORγt^+^ IL-17A^+^ cells. (J) IL-17A concentrations in matched culture supernatants measured by ELISA. Data are presented as median ± interquartile range from *n* = 6 donors. Statistical significance was assessed using the Friedman repeated-measures test with Dunn post hoc correction (*P < 0.05, **P < 0.01). Gates were defined using fluorescence-minus-one controls and applied uniformly across all samples.

To determine how progestins influence development of helper T cells (CD4^+^ T cells), we pretreated naïve CD4^+^ T cells with P4, levonorgestrel, or drospirenone. The cells were then stimulated with αCD3 and αCD28 for 24 hours followed by lineage-specific cytokine cocktails to induce Th1, Th2, or Th17 differentiation (Fig. 7A).^32^ After 7 days of culture, transcription factor and cytokine co-expression were assessed using flow cytometry to quantify lineage commitment. Routine sorting consistently yielded >95% naïve CD4^+^ T-cell purity, and successful polarization into Th0, Th1, Th2, and Th17A subsets was confirmed by cytokine ELISA (Fig. S7A–E). The gating strategy used for these analyses is shown in Fig. S8.

P4 exerted a clear ability to regulate Th cell differentiation. In Th1 differentiating cultures, P4 significantly reduced frequencies of IFN-γ^+^T-bet^+^ cells compared to media control (Fig. 7B and C), indicating ability of P4 to prevents cells from becoming the proinflammatory Th1 phenotype. Consistent with this, IFN-γ protein levels were significantly decreased in P4-treated cultures (Fig. 7D, *P* < 0.05). In contrast, neither levonorgestrel nor drospirenone altered Th1 polarization, suggesting that the suppressive effect on Th1 differentiation is specific to P4 rather than a shared property of all progestins.

Additionally, P4 promoted the Th2 lineage commitment. The frequency of IL-4^+^GATA3^+^ cells was significantly increased in P4-treated cultures (Fig. 7E and F, *P* < 0.01), demonstrating enhanced Th2 polarization. Consistent with this shift, IL-4 protein levels measured were also elevated relative to the media control (Fig.  7G), indicating that P4 not only increases Th2 commitment but also enhances downstream IL-4 production. Levonorgestrel and drospirenone again showed no measurable effect on Th2 differentiation.

Th17 differentiation remained uniformly low across all conditions. Frequencies of RORγt^+^IL-17A^+^ cells did not differ significantly between treatment groups (Fig. 7H and I), and IL-17A secretion was similarly unchanged (Fig. 7J). These findings indicate that neither P4 nor synthetic progestins substantially influence Th17 lineage commitment under these culture conditions. We also analyzed the effects of progesterone/progestins in Th0 cells without any polarizing conditions and observed that steroid treatments did not alter cytokine output under Th0 conditions, as frequencies of T-bet^+^IFN-γ^+^, GATA3^+^IL-4^+^, and RORγt^+^IL-17A^+^ cells remained comparable across all treatment groups (Fig. S9A–F).

Together, these data demonstrate that P4 selectively suppresses Th1 differentiation while enhancing Th2 polarization, consistent with its known immunoregulatory role.^38–41^ In contrast, levonorgestrel and drospirenone do not have this regulatory effect, indicating that synthetic progestins do not recapitulate the T-cell–modulating properties of P4.

## Discussion

This study demonstrates that commonly used selected synthetic progestins showed compound-specific immunological effects in primary human immune cells. Using physiologically relevant concentrations, we show that selected second and fourth-generation progestins, particularly levonorgestrel and drospirenone, induce elevated inflammatory cytokine production in PBMCs from healthy female donors. These effects were characterized by increased IL-6 secretion, broader activation of pro- and anti-inflammatory cytokine networks, preferential activation of monocyte populations, and altered Th differentiation profiles. In contrast, natural progesterone (P4) exhibited a comparatively immunoregulatory phenotype, suppressing Th1 differentiation and promoting Th2 lineage commitment without inducing inflammatory cytokine release.^40^^,^^42^^,^^43^

Among all cytokines examined, IL-6 emerged as the most consistently induced mediator following synthetic progestin exposure. Although P4 induced modest IL-6 responses in a subset of donors, levonorgestrel, norgestrel, norgestimate, and drospirenone produced stronger and more consistent IL-6 induction, particularly at higher concentrations.

IL-6 is a pleiotropic cytokine with well-established roles in innate immune activation, acute phase responses, and downstream T-cell polarization.^33^^,^^44^^,^^45^ Elevated IL-6 has also been implicated in chronic inflammatory and autoimmune conditions, including MS, rheumatoid arthritis, and systemic lupus erythematosus.^46–52^ The preferential induction of IL-6 by synthetic progestins suggests that structural modifications present in later-generation progestins may selectively engage inflammatory signaling pathways not activated by P4. This concept aligns with clinical observations in progestogen hypersensitivity, where exposure to endogenous or exogenous progestins triggers exaggerated inflammatory responses including elevated IL-6 in sensitized individuals.^53^^,^^54^

Importantly, the proinflammatory effects observed here extended beyond IL-6 alone and were supported at both the protein and transcriptional levels. Synthetic progestins induced IL-1β and GM-CSF in a compound-specific manner, with levonorgestrel producing the strongest IL-1β responses and norgestimate preferentially enhancing GM-CSF production. GM-CSF, in particular, has gained attention as a key mediator of pathogenic myeloid activation and neuroinflammation.^55^^,^^56^ These findings were further supported by qPCR analyses, where levonorgestrel and drospirenone significantly increased IL-6 transcription and levonorgestrel robustly upregulated IL-1β mRNA expression. In contrast, drospirenone produced only modest IL-1β transcriptional induction, paralleling its more limited IL-1β protein response. GM-CSF transcriptional responses were more pronounced than corresponding protein secretion, particularly for drospirenone, suggesting that transcriptional activation may precede or exceed detectable cytokine release under short-term exposure conditions. Notably, TNF-α remained largely unchanged at both the mRNA and protein levels, indicating that not all inflammatory pathways are equally responsive to progestin exposure. Interestingly, although synthetic progestins preferentially promoted proinflammatory cytokine responses, all compounds tested significantly induced IL-10 transcription at higher concentrations. However, only P4 consistently increased IL-10 protein secretion, whereas synthetic progestins failed to induce comparable IL-10 release despite elevated mRNA expression. This divergence between IL-10 transcription and protein secretion may reflect posttranscriptional regulation, delayed cytokine translation or secretion kinetics, or insufficient activation of the broader regulatory pathways required for sustained anti-inflammatory cytokine production. In contrast to the pronounced induction of IL-6, IL-1β, and GM-CSF, TGF-β levels remained stable across treatment conditions. Together, these findings demonstrate that synthetic progestins selectively amplify proinflammatory immune signaling while failing to fully reproduce the balanced immunoregulatory effects of P4, thereby supporting the concept that structurally distinct progestins can differentially shape innate immune responses in human PBMCs.

Among the receptor–cytokine associations identified, baseline GCR expression showed the strongest association with progesterone-associated IL-6 responses. Although classical GCR signaling is generally linked to anti-inflammatory activity and suppression of IL-6 production, glucocorticoid signaling can exhibit context-dependent immunomodulatory effects influenced by receptor cross-talk, ligand availability, cellular activation state, and interactions with other steroid receptor pathways.^57–59^ Because receptor expression alone did not consistently predict cytokine responsiveness across donors, these findings should be interpreted cautiously and viewed as exploratory rather than mechanistic. Together, these data suggest that downstream signaling properties and broader steroid receptor network interactions may contribute more substantially to inflammatory responsiveness than baseline receptor abundance itself.

Beyond the GCR-associated IL-6 findings, several additional receptor–cytokine relationships emerged that may provide insight into how synthetic progestins engage broader steroid signaling pathways. Norgestimate-induced IL-1β secretion positively correlated with baseline PR expression, whereas progesterone-associated GM-CSF responses correlated with mPR, AR, and MR expression. Drospirenone-induced GM-CSF secretion correlated with PR, GCR, and AR expression, and drospirenone-associated TNF-α responses showed a modest positive association with GCR expression. Collectively, these findings support the possibility that synthetic progestins engage interconnected androgenic, glucocorticoid, mineralocorticoid, and mPR signaling pathways rather than acting exclusively through classical PR signaling. Donor age also showed no meaningful association with IL-6, IL-1β, GM-CSF, or TNF-α production, reinforcing that demographic factors do not substantially shape inflammatory responsiveness to natural or synthetic progestins in this cohort. Overall, these data suggest that the heightened inflammatory activity of synthetic progestins is more likely driven by compound-specific signaling properties and cross-talk within the broader steroid receptor network, rather than by baseline receptor abundance or donor age.

Several population-based studies have reported associations between hormonal contraceptive use and increased incidence of immune-mediated diseases, including MS.^13^^,^^14^ While such associations do not establish causality and may reflect complex behavioral or prodromal confounding factors, the inflammatory responses observed in this study provide biological plausibility for how synthetic progestins could tilt the balance toward immune priming in susceptible individuals. The IL-6 dominant signature observed here is particularly notable, as IL-6 signaling is increasingly recognized as a central driver of autoimmune pathogenesis and disease progression.^47^^,^^49^^,^^52^^,^^60^ These findings do not imply that progestins universally promote disease development. Rather, they suggest that in certain immune contexts or genetically predisposed individuals, synthetic hormone exposure may modulate inflammatory thresholds in a manner that warrants further investigation.

A critical strength of this study is the use of hormone concentrations that approximate reported circulating levels following clinical administration. Many prior in vitro studies examining steroid hormone effects have relied on supraphysiologic micromolar concentrations that exceed realistic exposure ranges and may produce nonspecific cellular stress responses.^61^^,^^62^ In contrast, we observed consistent transcriptional and protein-level cytokine induction at nanogram per milliliter concentrations that overlap with clinically measured peak serum levels. This suggests that the inflammatory effects reported here are not artifacts of pharmacologic overdose but may reflect biologically relevant immune modulation.

High-dimensional single-cell analysis and conventional flow cytometry gating converged on CD14^+^ monocytes as the dominant IL-6 producing population following synthetic progestin exposure. Monocytes express multiple steroid receptors and play central roles in cytokine amplification, antigen presentation, and orchestration of adaptive immune responses.^63–66^ Previous studies have demonstrated that P4 can suppress monocyte activation, whereas selected synthetic progestins may fail to reproduce this regulatory effect.^62^^,^^67–69^ Our findings extend this work by identifying monocytes as the principal cellular target responsible for progestin-associated IL-6 induction in mixed immune populations. The magnitude of monocyte activation was higher with levonorgestrel and drospirenone exposure, consistent with its broad receptor engagement profile observed in our receptor expression analysis. Notably, aberrant monocyte activation has also been implicated in progestogen hypersensitivity disorders, in which synthetic progestins trigger exaggerated cytokine responses and systemic inflammatory symptoms.^53^ Together, these results suggest that innate immune reprogramming may represent an early and central mechanism through which synthetic progestins shape downstream immune outcomes.

Natural progesterone selectively suppressed Th1 differentiation and promoted Th2 lineage commitment, consistent with its established role in maintaining immune tolerance during pregnancy and limiting excessive inflammatory responses.^67^^,^^70^ In contrast, levonorgestrel and drospirenone failed to reproduce these regulatory effects. The absence of Th1 suppression by synthetic progestins may allow persistence of inflammatory T-cell phenotypes in contexts where progesterone would normally exert immunoregulatory control. Interestingly, neither progesterone nor synthetic progestins significantly altered Th17 differentiation under our experimental conditions. This suggests that the observed inflammatory effects are driven primarily by innate immune activation and altered Th1/Th2 balance rather than direct promotion of Th17 polarization. These findings parallel evidence from neural systems showing that certain synthetic progestins can actively interfere with progesterone-mediated protective signaling. In neurons, medroxyprogesterone acetate fails to reproduce progesterone’s neuroprotective actions and instead antagonizes estrogen- and progesterone-induced MAPK signaling, thereby blocking key pathways required for neuronal survival and metabolic support.^71–73^ This divergence between P4 and synthetic progestins in the central nervous system mirrors our observations in primary human T cells, where levonorgestrel and drospirenone failed to suppress Th1 differentiation or promote Th2 commitment, in contrast to the robust immunoregulatory phenotype induced by progesterone. Further investigation is warranted to delineate the specific signaling cascades activated within individual PBMC subsets, particularly monocytes and T cells, to determine how synthetic progestins reprogram innate and adaptive immune pathways at the molecular level.

In summary, our findings demonstrate that selected synthetic progestins, particularly levonorgestrel and drospirenone, induce inflammatory cytokine production in primary human immune cells at physiologically relevant concentrations, while P4 maintains immunoregulatory effects on Th differentiation. These responses are driven primarily by monocyte activation and are associated with broad modulation of steroid receptor expression.

Although the present study identifies distinct inflammatory effects of selected synthetic progestins, several limitations should be considered when interpreting these findings. First, the immune responses observed were heterogeneous across individual progestins, cytokines, and donors, indicating that inflammatory activation is not a universal feature of all synthetic progestins. While levonorgestrel and drospirenone consistently demonstrated stronger inflammatory signatures, other compounds produced more modest or selective effects depending on the cytokine measured. A subset of donors also appeared nonresponsive across one or more cytokines. This likely reflects intrinsic heterogeneity in steroid receptor signaling, baseline immune activation state, posttranscriptional regulation, and assay detection limits rather than a true absence of biological responsiveness. Donors differ in receptor isoform composition, chromatin accessibility, co-regulator availability, and tonic inflammatory tone, any of which can limit the ability of progestins to drive detectable cytokine secretion in short-term PBMC assays. Second, this study focused on short-term PBMC exposure assays in healthy donors and therefore does not fully capture the complexity of chronic in vivo hormonal exposure, tissue-specific immune regulation, endogenous hormonal cycling, or interactions with additional immune and stromal cell populations. Finally, while the observed correlations between baseline receptor mRNA levels and cytokine secretion highlight potential donor-specific patterns, these relationships are purely associative and do not indicate receptor activation, signaling engagement, or functional involvement. Further mechanistic studies using purified immune subsets, receptor-specific perturbation approaches, and in vivo models will be necessary to define how structurally distinct synthetic progestins differentially regulate immune responses.

Importantly, this study does not establish causality between progestin use and inflammatory disease development. Rather, it identifies potential immunological mechanisms that may contribute to differential immune responses among individuals exposed to synthetic hormones. Larger population-based studies across diverse geographic regions and hormonal formulations will be required to determine whether specific subsets of individuals exhibit heightened inflammatory sensitivity to progestin exposure. A more detailed understanding of these mechanisms may ultimately enable personalized contraceptive strategies that account for individual immune responsiveness, genetic background, and inflammatory risk profiles. Such approaches could improve both safety and efficacy while minimizing unintended immune effects.

## Supplementary Material

vlag046_Supplementary_Data

## Data Availability

The data underlying this article will be shared on reasonable request to the corresponding author.
