# Supplementary material for: Distinct immunomodulatory effects of synthetic progestins compared with natural progesterone in human immune cells
Source: Immunohorizons. 2026 Aug 17;10(8):vlag046. doi: 10.1093/immhor/vlag046 (PMC13480475; doi:10.1093/immhor/vlag046)
Supplement: Supplementary file 1 [file vlag046_supplementary_data.zip › Supplement_Data/Supplemental Figure_07.06.26.docx]

**Figure S1: Optimization of culture conditions for PBMC stimulation. A** Cell seeding density optimization following stimulation of PBMCs with 1, 10, and 100 pg/mL LPS, with IL‑6 secretion quantified by ELISA to determine the density yielding maximal dynamic range. **B** Dose–response curves for IL‑6, TNFα, IFNγ, and IL‑1β production after stimulation with 1, 10, and 100 pg/mL LPS, identifying optimal concentrations of LPS for downstream assays. **C** Summary table outlining optimal stimulation conditions for additional cytokines under LPS and PMA/Ionomycin activation.

**Figure S2: Correlation between baseline receptor mRNA expression and IL-1b production following progestin treatment.** Baseline mRNA expression of progesterone receptor (PR), membrane progesterone receptor (mPR), glucocorticoid receptor (GCR), androgen receptor (AR), mineralocorticoid receptor (MR), and estrogen receptor alpha (ERα) was correlated with IL‑1β secretion (pg/ml) after treatment with progesterone, levonorgestrel, or norgestimate (50 ng/ml). Each panel shows individual donor values (n=20) with a fitted line and 95% confidence interval. Reported statistics are Spearman correlation coefficients (ρ) with corresponding P values; the r² values shown in the panels represent Spearman rho (ρ). **A to C** show correlations for PR, **D to F** for mPR, **G to I** for GCR, **J to L** for AR, **M to O** for MR, and **P to R** for ERα under progesterone, levonorgestrel, and drospirenone treatment, respectively.

Baseline Receptor mRNA expression

**Figure S3: Correlation between baseline receptor mRNA expression and GM-CSF production following progestin treatment.** Baseline mRNA expression of progesterone receptor (PR), membrane progesterone receptor (mPR), glucocorticoid receptor (GCR), androgen receptor (AR), mineralocorticoid receptor (MR), and estrogen receptor alpha (ERα) was correlated with GM‑CSF secretion (pg/ml) after treatment with progesterone, levonorgestrel, or drospirenone (50 ng/ml). Each panel shows individual donor values (n =20) with a fitted line and 95% confidence interval. Reported statistics are Spearman correlation coefficients (ρ) with corresponding P values; the r² values shown in the panels represent Spearman rho (ρ). A-C show correlations for PR, D-F for mPR, G-I for GCR, J-L for AR, M-O for MR, and P-R for ERα under progesterone, levonorgestrel, and drospirenone treatment, respectively.

**Figure S4: Correlation between baseline receptor mRNA expression and TNFα production following progestin treatment.** Baseline mRNA expression of progesterone receptor (PR), membrane progesterone receptor (mPR), glucocorticoid receptor (GCR), androgen receptor (AR), mineralocorticoid receptor (MR), and estrogen receptor alpha (ERα) was correlated with TNFα secretion (pg/ml) after treatment with progesterone, levonorgestrel, or drospirenone (50 ng/ml). Each panel shows individual donor values (n=20) with a fitted line and 95% confidence interval. Reported statistics are Spearman correlation coefficients (ρ) with corresponding P values; the r² values shown in the panels represent Spearman rho (ρ). A to C show correlations for PR, D to F for mPR, G to I for GCR, J to L for AR, M to O for MR, and P to R for ERα under progesterone, levonorgestrel, and drospirenone treatment, respectively.

**Figure S5:  Correlation between age and cytokine production following hormone treatment.** Age (years) was correlated with IL‑1β, GM‑CSF, and TNFα secretion (pg/ml) after treatment with progesterone, norethindrone, norgestrel, levonorgestrel, norgestimate, or drospirenone (50 ng/ml). Each panel shows individual donor values (n =20) with a fitted line and 95% confidence interval. Reported statistics are Spearman correlation coefficients (ρ) with corresponding P values; the r² values shown in the panels represent Spearman rho (ρ). A to C show correlations for progesterone, D to F for norethindrone, G to I for norgestrel, J to L for levonorgestrel, M to O for norgestimate, and P to R for drospirenone across IL‑1β, GM‑CSF, and TNFα, respectively.

**Figure S6:  Gating strategy for identifying IL‑6–producing cell populations in bulk PBMCs.** Sequential gating was performed to define major PBMC lineages and quantify IL‑6–producing cells within each subset. Gates were established using fluorescence‑minus‑one (FMO) controls to ensure accurate discrimination of IL‑6⁺ events.

**Figure S7. Purity of naïve CD4⁺ T cell isolation and validation of Th lineage polarization.** **A-B**. Flow cytometry plots showing pre- and post-sort purity of naïve CD4⁺ T cells isolated from PBMCs using magnetic bead enrichment for CD45RA and CD4. Post-sort purity of CD4⁺CD45RA⁺ cells exceeded 94%, as determined by quadrant gating. **C-E** Cytokine production following polarization under Th0, Th1, Th2, and Th17A conditions. **C** IFNγ levels confirm Th1 skewing. **D** IL-4 levels confirm Th2 polarization. E IL-17A levels confirm Th17A differentiation.

**A**

**B**

**C**

**D**

**E**

**Figure S8: Flow cytometric gating strategy for Th1, Th2, and Th17A subsets**. Live, single lymphocytes were gated from PBMCs and refined to CD3⁺CD4⁺ T helper cells. Subsets were identified based on canonical transcription factors and intracellular cytokines: T-bet/IFNγ for Th1, GATA3/IL-4 for Th2, and RORγ/IL-17A for Th17A. Gating thresholds were defined using fluorescence-minus-one (FMO) controls.

**Figure S9. Flow cytometric analysis of Th0 cells following hormonal treatment.** Naïve CD4⁺ T cells (n = 6) were pre‑incubated with steroids (media, progesterone [P4], levonorgestrel, or drospirenone) for 24 hours, followed by activation with plate‑bound anti‑CD3 and anti‑CD28 under non‑polarizing Th0 conditions. Cultures were maintained for 7 days, with hormones refreshed on day 3. On day 7, cells were restimulated with PMA/ionomycin in the presence of brefeldin A for 4 hours prior to intracellular staining. CD4⁺ T cells were gated from live, single, CD3⁺ lymphocytes and analyzed for lineage‑associated transcription factor and cytokine expression. **(A-B)** Th1‑associated phenotype identified by T‑bet and IFN‑γ expression. **(C-D)** Th2‑associated phenotype identified by GATA3 and IL‑4 expression. **(E-F)** Th17‑associated phenotype identified by RORγt and IL‑17A expression. Statistical comparisons across treatments were performed using the Friedman test with Dunn’s post‑hoc correction. A p value < 0.05 was considered statistically significant.
