# Supplementary material for: Distinct immunomodulatory effects of synthetic progestins compared with natural progesterone in human immune cells
Source: Immunohorizons. 2026 Aug 17;10(8):vlag046. doi: 10.1093/immhor/vlag046 (PMC13480475; doi:10.1093/immhor/vlag046)
Supplement: Supplementary file 1 [file vlag046_supplementary_data.zip › Supplement_Data/Supplemental_Tables_7.6.26.docx]

**Table S1: List of primers used for the study**

| Gene | Forward | Reverse | Amplicon Size |
| --- | --- | --- | --- |
| *IL-6* | ACTCACCTCTTCAGAACGAATTG | CCATCTTTGGAAGGTTCAGGTTG | 149 |
| *IL-1β* | ATGATGGCTTATTACAGTGGCAA | GTCGGAGATTCGTAGCTGGA | 132 |
| *IFNγ* | TCGGTAACTGACTTGAATGTCCA | TCGCTTCCCTGTTTTAGCTGC | 93 |
| *TNFα* | GAGGCCAAGCCCTGGTATG | CGGGCCGATTGATCTCAGC | 91 |
| *GMCSF* | TCCTGAACCTGAGTAGAGACAC | TGCTGCTTGTAGTGGCTGG | 187 |
| *IL-10* | GACTTTAAGGGTTACCTGGGTTG | TCACATGCGCCTTGATGTCTG | 112 |
| *Progesterone receptor (PR)* | ACCCGCCCTATCTCAACTACC | AGGACACCATAATGACAGCCT | 133 |
| *Glucocorticoid receptors (GCR)* | ACAGCATCCCTTTCTCAACAG | AGATCCTTGGCACCTATTCCAAT | 99 |
| *Progesterone receptor membrane component 1 (mPR)* | GGGCTGCTGCATGAGATTTTC | CCGCGCACGATCTTGTAGA | 86 |
| *Androgen receptor (AR)* | CCAGGGACCATGTTTTGC | CGAAGACGACAAGATGGACAA | 226 |
| *Mineralocorticoid receptor (MR)* | GAAAGACGGTGGGGTCAAGTT | ACCGGAAACACAGCTTACGTT | 114 |
| *Estrogen receptor alpha (ERα)* | CCCACTCAACAGCGTGTCTC | CGTCGATTATCTGAATTTGGCC | 180 |

**Table S2: Naïve CD4 T cell Th polarization**

| Th subsets | aCD3+ aCD28 | Neutralizing Antibodies | Cytokines |
| --- | --- | --- | --- |
| Tho | + | - |  |
| Th1 | + | αIL-4 | IL-2 + IL-12 |
| Th2 | + | αIFNy | IL-2 + IL-4 |
| Th17 | + | αIL-4 + αIFNy | TGFβ + IL-6 + IL-1β |
